# Tokenization techniques for privacy-preserving healthcare data: tokenization nuts and bolts

**DOI:** 10.3389/fdsfr.2025.1599217

**Published:** 2025-12-18

**Authors:** Camille V. Cook

**Affiliations:** LexisNexis Risk Solutions, Alpharetta, GA, United States

**Keywords:** tokenization, real-world data (RWD), linking, privacy, pharmocovigilance, health economics

## Abstract

Tokenization is a crucial technology for ensuring the security and privacy of patient data in clinical research, pharmacovigilance, and drug safety monitoring. As healthcare increasingly integrates diverse data sources-ranging from clinical records to non-clinical data such as social determinants of health (SDOH)-it is essential to protect sensitive patient information while improving data quality and analysis (National Institutes of Health, 2006). This article emphasizes tokenization’s critical role in safeguarding privacy, particularly in pharmacovigilance activities including safety monitoring, risk assessment, and post-market surveillance. Beyond security, tokenization enriches research datasets by enabling integration of external information, thereby enhancing the rigor and reliability of pharmacovigilance outcomes. With effective tokenization, researchers can better protect patients while gaining deeper insights into clinical and pharmacological research (Cruz et al., 2024). Recent global applications validate tokenization as a foundational privacy-preserving technology in pharmacovigilance. An applied example from a psoriasis clinical trial demonstrated referential tokenization’s capacity to securely link electronic health records (EHRs) and claims data across systems with greater than 99% linkage precision while maintaining privacy standards (D'Andrea et al., 2024). These capabilities align with emerging international frameworks, including the European Health Data Space (2025), reinforcing tokenization’s value in generating regulatory-grade evidence for pharmacovigilance across national and multinational research environments. In many jurisdictions, tokenization that meets de-identification or pseudonymization standards may not require individual patient consent, though this varies based on data sensitivity, jurisdictional law, and the study’s intent (Office for Civil Rights, 2023; EDPB, 2021).

## 1 Introduction

The modern healthcare landscape increasingly relies on integrating heterogeneous datasets to advance clinical research and pharmacovigilance activities. Patient-level data—encompassing clinical observations, medication histories, laboratory results, and socio-environmental contexts—are critical for real-world evidence (RWE), personalized medicine, regulatory decision-making, and comprehensive pharmacovigilance. However, the sensitivity of personally identifiable information (PII) necessitates rigorous privacy protection. Tokenization addresses this imperative by substituting sensitive identifiers with pseudonymous tokens. While referential and salted tokenization methods are generally irreversible, deterministic or non-salted tokenization may permit re-identification under specific conditions (e.g., access to keys or mapping tables). Thus, tokenization’s irreversibility depends on implementation context and security controls (Bernstam et al., 2023; Yue, 2022).

Decentralized clinical trials, multi-institutional observational studies, and regulatory-grade safety surveillance increasingly rely on robust tokenization techniques to enable privacy-compliant linkage across diverse data sources—including electronic health records (EHRs), insurance claims, mortality registries, and emerging domains such as social determinants of health (SDOH) and genomics—supporting high-quality evidence generation for regulatory decision-making. This integration fosters comprehensive longitudinal patient profiles essential for pharmacovigilance, enabling detection of adverse drug reactions, safety signal validation, and the study of treatment pathways and health disparities (Cruz et al., 2024). Subsequent sections examine tokenization methodologies, workflows, and regulatory considerations fundamental to privacy-preserving data linkage for pharmacovigilance.

## 2 Methods of tokenization

### 2.1 Tokenization methodologies and their characteristics

Tokenization replaces direct identifiers—such as names, dates of birth, and social security numbers—with tokens that allow cross-dataset linkage without exposing raw PII. Three primary tokenization paradigms dominate:

1. Deterministic tokenization applies a consistent algorithm producing the same token for identical inputs, facilitating reproducible linkage across data sources. This method is highly effective for structured datasets with stable identifiers, enabling longitudinal patient tracking and outcome analyses vital for pharmacovigilance. However, deterministic tokens may be vulnerable to re-identification if underlying algorithms are reverse-engineered or keys compromised (Bernstam et al., 2023).
2. Randomized tokenization generates unique tokens upon each application, maximizing privacy through obfuscation. While reducing re-identification risk, randomized tokens preclude direct record linkage, limiting utility for longitudinal or multi-source data integration critical in pharmacovigilance. Thus, this method suits anonymized datasets intended for aggregate safety analyses or exploratory research where linkage is unnecessary (Bernstam et al., 2023).
3. Referential tokenization leverages a secure reference repository and cryptographic hashing of demographic elements and identifiers to generate tokens. By abstracting identifiers into irreversible pseudonyms and managing mappings within secure environments, referential tokenization enables precise, privacy-compliant linkage of heterogeneous datasets. This approach is widely adopted for regulatory-grade pharmacoepidemiology and pharmacovigilance studies, supporting integration of EHR, claims, mortality, and SDOH data at scale (D’Andrea et al., 2024; Eckrote et al., 2024).

Referential tokenization’s scalability and accuracy have been demonstrated in real-world applications, such as the linkage of large payer and non-payer datasets, with linkage precision exceeding 99% in high-fidelity settings (D’Andrea et al., 2024). This level of performance facilitates comprehensive safety signal detection, benefit-risk assessment, and population health research essential to pharmacovigilance.

Note: The overlap with non-payer (open) claims data was approximately 90%, based on linkage to clearinghouse data representing >70 million lives. By contrast, overlap with payer-specific datasets was ∼40%, though with higher precision in capturing longitudinal medical claims.

Table 1 summarizes the three primary tokenization methods alongside their key advantages for pharmacovigilance and pharmacoepidemiology.

**TABLE 1:** Tokenization methods and their applications.

Tokenization method	Description	Advantages in pharmacovigilance and pharmacoepidemiology
Deterministic	Generates a fixed token for each data element, ensuring that the same input data always produces the same token. Ideal for structured data integration, ensuring consistent and reliable data linkage.	Enables longitudinal tracking of patient outcomes; efficient analysis across datasets.
Randomized	Generates a different token each time for the same data element, increasing privacy by obscuring relationships. This makes it more difficult to infer useful information from tokenized data but complicates data queries and may reduce match rates across datasets.	Enhances patient privacy; prevents re-identification, especially in real-world data studies.
Referential	Combines deterministic and randomized tokenization. Uses tokens referring to an external “reference” or lookup table, balancing flexibility and privacy. Ensures high match rates between datasets, making it ideal for integrating external data sources.	Allows secure, dynamic access to original data for analysis; flexible for cross-dataset linkage.

### 2.2 Tokenization workflow and global regulatory framing

A robust tokenization workflow typically involves several key stages that ensure privacy preservation in pharmacovigilance as seen in Table 2:

1. Identification of Sensitive Data Elements: Initially, data custodians identify direct identifiers (e.g., patient names, medical record numbers, exact dates of birth) and quasi-identifiers (e.g., ZIP codes, gender) subject to privacy protection (Bernstam et al., 2023).
2. Token Generation: Cryptographic algorithms, often based on Secure Hash Algorithm (SHA) variants, produce fixed-length tokens that are deterministic and non-reversible without access to secure keys or lookup tables (Yue, 2022; Eckrote et al., 2024). The selection of algorithmic parameters is critical to balancing linkage fidelity and privacy.
3. Secure Mapping and Storage: The token-to-identifier mappings are stored separately in encrypted, access-controlled repositories, minimizing risks of data leakage. Best practices mandate AES-256 encryption and stringent role-based access controls to enforce separation of duties (Walters et al., 2025).
4. Linkage and Translation Across Datasets: Tokenized identifiers enable “cross-walking” between datasets originating from disparate systems, often with heterogeneous data standards and terminologies. Referential tokenization supports the use of a “golden record” as a canonical reference to resolve discrepancies and enhance matching accuracy (Dunne, 2023).
5. Data Enrichment: Tokenized datasets can be securely enriched with auxiliary data streams such as SDOH, genomic profiles, and mortality records, adding contextual layers that enhance RWE analyses without compromising privacy (Li et al., 2024; Elhussein et al., 2024).

**TABLE 2:** Tokenization workflow.

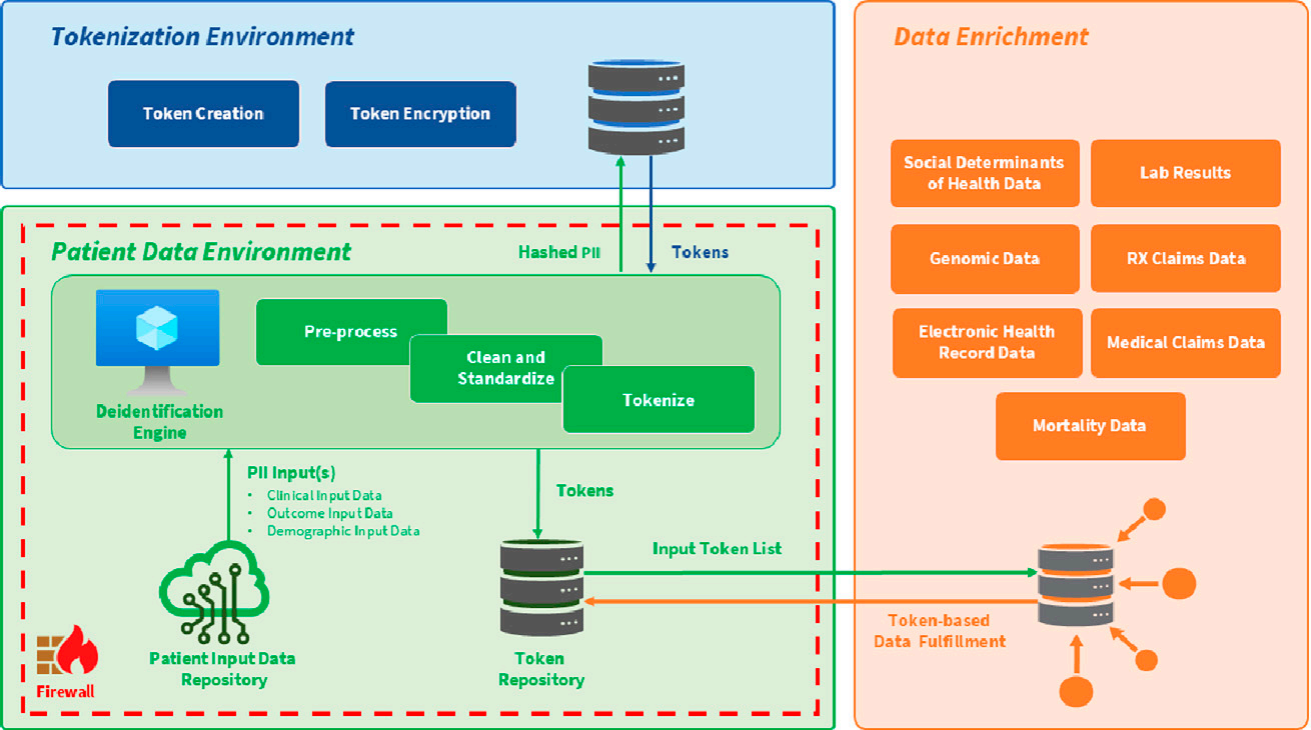

The diagram above shows the process of identifying sensitive data, generating and securely storing tokens, enabling their use while maintaining privacy, managing the token lifecycle for compliance, and enriching the dataset with secondary data elements.

### 2.3 Regulatory context and global privacy frameworks

The adoption of tokenization is tightly linked to regulatory frameworks governing patient privacy and pharmacovigilance. The Health Insurance Portability and Accountability Act (HIPAA) in the United States, the General Data Protection Regulation (GDPR) in the European Union, and the European Health Data Space Regulation (EU 2025/327) collectively emphasize pseudonymization and de-identification as cornerstones for lawful data reuse in safety surveillance (European Parliament, 2025; European Data Protection Board, 2021; Office for Civil Rights, 2023).

Referential tokenization aligns with these frameworks by enabling deterministic linkage via pseudonymous tokens while preventing the exchange or exposure of PII. The EHDS explicitly endorses token-based data linkage for secondary use of health data across EU member states, contingent upon compliance with GDPR principles (European Commission, 2025). Such alignment facilitates multinational clinical trials and pharmacovigilance studies that require longitudinal datasets while respecting data sovereignty and patient rights.

## 3 Technical challenges

### 3.1 Data variability and patient-centricity

Clinical trial and pharmacovigilance datasets are inherently dynamic, reflecting ongoing changes in patient health status, treatment responses, and personal circumstances (Li et al., 2024). Tokenization frameworks must account for this variability to preserve critical longitudinal information needed for personalized interventions and accurate safety assessments. Failure to accommodate these fluctuations risks oversimplifying patient trajectories, diminishing the clinical and scientific value of pharmacovigilance data. Overreliance on aggregated population-level data risks masking heterogeneity in treatment effects and adverse event profiles, which are essential considerations in pharmacovigilance and precision medicine (Abul-Husn and Kenny, 2019). Consequently, tokenization methodologies should maintain sufficient granularity to support nuanced, patient-centric pharmacovigilance analyses throughout the clinical research lifecycle.

### 3.2 Linkage precision: balancing over-linking and under-linking

Accurate linkage of tokenized data is fundamental in pharmacovigilance and health economic studies. Over-linking, when unrelated patient records are mistakenly connected, can introduce confounding bias by falsely aggregating clinical events, exposures, or outcomes across distinct individuals. This misattribution may distort incidence rates, mask true safety signals, or generate spurious associations, ultimately compromising the validity of pharmacovigilance and real-world evidence analyses (Velummailum et al., 2023). Conversely, under-linking—failure to match related records—results in incomplete patient profiles and potential missed adverse drug reactions or treatment outcomes. Referential tokenization employs advanced matching algorithms supported by “golden records” or trusted data sources to mitigate these risks, enhancing the precision of pharmacovigilance cross-dataset linkage and minimizing both false positives and false negatives (Dunne, 2023).

### 3.3 Data harmonization and integrity

Tokenization’s effectiveness depends heavily on the harmonization of data across diverse sources with varying standards, formats, and completeness (Velummailum et al., 2023). Clinical trial and real-world pharmacovigilance data often originate from heterogeneous environments, complicating direct linkage. Without rigorous data preprocessing and standardization, tokenization can generate mismatches or fragmentations, threatening data integrity and skewing pharmacovigilance outcomes (Walters et al., 2025). Incorporating privacy-preserving record linkage (PPRL) mechanisms alongside tokenization further ensures secure integration of datasets without exposing sensitive patient identifiers. However, weak encryption or lax access controls risk breaching patient confidentiality, undermining trust in the entire pharmacovigilance data ecosystem.

### 3.4 Linking Economical and clinical data

In health economics and safety studies, linking clinical trial and pharmacovigilance data to economic datasets is pivotal for accurate cost-effectiveness and safety analyses. Inaccuracies in tokenization or linkage processes can yield incomplete or biased datasets, obscuring critical cost drivers or patient-level treatment responses. This threatens the reliability of economic evaluations that inform reimbursement and regulatory decisions, potentially affecting trial feasibility and therapeutic innovation (Li et al., 2024).

### 3.5 Integrating primary and secondary data sources

Merging primary clinical trial and pharmacovigilance datasets with secondary sources—such as claims, social determinants of health (SDOH), genomic, and mortality data—introduces additional complexities. The distinction between primary and secondary data depends on collection context. Primary data refers to information collected for the specific study at hand (e.g., clinical trial data), whereas secondary data (e.g., claims or SDOH) is repurposed for analysis. Depending on how pharmacovigilance data is generated, it may fall into either category (Gliklich et al., 2014). These datasets often differ in structure and standards, necessitating robust preprocessing and referential matching algorithms to ensure consistent token mapping across all sources (Eckrote et al., 2024). Failure to properly align these data can degrade the accuracy of pharmacovigilance real-world evidence (RWE) and impede valid interpretation. Furthermore, enriching tokenized datasets with external data must be carefully managed within regulatory frameworks to prevent inadvertent exposure of protected health information (Office for Civil Rights, 2023).

### 3.6 Controlled re-identification for safety signal follow-up

A critical challenge involves enabling controlled, secure re-identification for pharmacovigilance activities such as safety signal validation and adverse event follow-up. Tokenization frameworks in pharmacoepidemiology often include tightly regulated re-identification protocols managed by neutral data custodians. These custodians maintain encrypted mappings between tokens and original identifiers within secure environments, allowing re-identification only under ethically approved, regulatorily compliant conditions (ENCePP, 2020). This approach balances stringent privacy protection with the operational necessity of patient-level follow-up in pharmacovigilance, supported by access controls, audit trails, and regulatory oversight.

### 3.7 Applied scenarios and criteria for tokenization in pharmacoepidemiology and safety surveillance

Although tokenization is gaining adoption in regulatory-grade data linkage, explicit examples in published pharmacoepidemiology studies are limited due to commercial and privacy constraints. The following three applied scenarios illustrate how tokenization enables privacy-preserving linkage for post-market safety research and real-world evidence (RWE) generation:

Scenario 1: Linking Claims and EHRs to Detect Adverse Events Post-Approval.

After FDA approval of a new anticoagulant, a manufacturer initiates post-marketing surveillance. Using referential tokenization, patient-level claims data (hospitalizations, diagnoses, procedures) are linked to EHRs (lab values, medication reconciliation, bleeding risk scores). This enables early detection of adverse drug reactions (e.g., GI bleeding) that may not be fully captured in claims alone fulfilling a regulatory risk minimization requirement (Eckrote et al., 2024).

- Tokenization Use: Demographic fields (e.g., full DOB, sex, ZIP code) are cryptographically hashed and tokenized by a third-party tokenization network.
- Outcome: Combined datasets support a retrospective cohort study on bleeding events stratified by renal function, fulfilling a regulatory risk minimization requirement.

Scenario 2: Linking Registry and Claims Data for Long-Term Drug Safety Monitoring.

A national rheumatoid arthritis (RA) registry collects longitudinal clinical data on disease activity, biologic use, and side effects. Researchers seek to evaluate long-term malignancy risk associated with a specific TNF inhibitor informing black-box warning updates (D’Andrea et al., 2024).

- Tokenization Use: Patients in the registry are tokenized using referential methods. Claims data from Medicare and commercial payers are then linked to capture incident cancer diagnoses, procedures, and prescription fills.
- Outcome: The linked dataset allows evaluation of rare, long-latency adverse events over multi-year periods, providing evidence for regulatory signal refinement and black-box warning updates.

Scenario 3: Linking Clinical Trial Participants to Claims for Safety Follow-up.

A pivotal oncology trial identifies a rare cardiovascular signal in interim analysis. To assess whether this signal emerges in broader populations, the sponsor uses tokenization to link trial participants to U.S. claims data post-trial enabling regulatory submission of updated safety data (DIA RWE, 2023).

- Tokenization Use: At consent, participant demographics are tokenized. Tokens are matched with commercial claims data over a 2-year period post-trial.

Outcome: Analysis identifies real-world incidence rates of cardiac events, enabling regulatory submission of updated safety data and refined risk communication in product labeling.

Table 3 summarizes pharmacoepidemiology-specific scenarios underscore tokenization’s pivotal role in enabling safety signal detection, longitudinal outcomes research, and regulatory-grade data integration—without compromising patient privacy. As tokenized infrastructures mature, their adoption will expand across global post-marketing surveillance networks and distributed data networks.

**TABLE 3:** Minimum criteria for tokenization in pharmacoepidemiology safety studies.

Category	Minimum requirement
Identifier Inputs	Full DOB, ZIP code, sex/gender, first & last name preferred (if available).
Tokenization Method	Referential tokenization with SHA-2 or SHA-3 algorithms, salted, non-reversible hashes. *Salted: Salted tokens involve adding a random value (a “salt”) to the identifier before hashing, improving security by preventing reverse-engineering of the token.*
Linkage Environment	Clean room or enclave with enforced access controls, audit logs, and role-based permissions. *Clean room or enclave: A highly secure computing environment that enforces role-based access, audit logs, and strict governance for analyzing sensitive data without exposing identifiable elements.* See Walters et al., 2025.
Linkage Accuracy	Match precision ≥95% validated via gold-standard test sets or sample clerical review.
Re-identification Protocols	Restricted to neutral data custodian under IRB- or regulator-approved protocol.
Regulatory Alignment	Must comply with HIPAA Expert Determination or GDPR pseudonymization guidelines.

## 4 Discussion

### 4.1 Conclusion: optimizing tokenization for clinical trial applications

In conclusion, tokenization serves as an essential enabler for privacy-preserving data integration in clinical research and pharmacovigilance. By securely replacing sensitive patient identifiers with irreversible tokens, tokenization enables comprehensive analysis of complex, multi-source datasets—ranging from electronic health records (EHRs) and claims to genomic and patient-reported outcomes—while maintaining patient confidentiality throughout the clinical trial and post-market pharmacovigilance lifecycle.

Referential tokenization, in particular, supports scalable, high-precision linkage of disparate datasets, ensuring compliance with evolving global privacy frameworks such as GDPR and the European Health Data Space (EHDS). This approach facilitates the construction of richer, longitudinal patient profiles essential for safety monitoring, benefit-risk assessment, and health equity research, all while minimizing the risk of data exposure through adherence to de-identification and pseudonymization standards.

To fully realize tokenization’s transformative potential in pharmacovigilance, key technical challenges must be addressed. These include ensuring data integrity through rigorous standardization and harmonization, managing token lifecycles effectively, maintaining linkage accuracy to avoid both over-linking and under-linking, and implementing controlled re-identification mechanisms for authorized pharmacovigilance follow-up.

Additionally, enriching tokenized datasets with secondary sources such as claims, social determinants of health (SDOH), and mortality data offers valuable clinical insights but demands strict governance to uphold data alignment and regulatory compliance.

Empirical evidence underscores tokenization’s impact, as demonstrated in applied clinical trial settings with secure linkage of real-world data achieving high precision, enabling robust longitudinal analyses and international collaboration under stringent privacy constraints (D’Andrea et al., 2024). As clinical research and pharmacovigilance increasingly rely on interoperable real-world data, tokenization will remain central to unlocking the full analytic value of healthcare datasets while safeguarding patient privacy.

Future directions should focus on refining tokenization algorithms, establishing international standards for tokenized data reporting, and advancing trusted data custodian models that balance patient confidentiality with investigational imperatives. These efforts will underpin the continued evolution of clinical research toward resilient, patient-centric paradigms that harmonize privacy preservation with data-driven innovation and regulatory rigor.
