# Supplementary figures and images for: Tokenization techniques for privacy-preserving healthcare data: tokenization nuts and bolts
Source: Front Drug Saf Regul. 2025 Dec 18;5:1599217. doi: 10.3389/fdsfr.2025.1599217 (PMC12756134; doi:10.3389/fdsfr.2025.1599217)

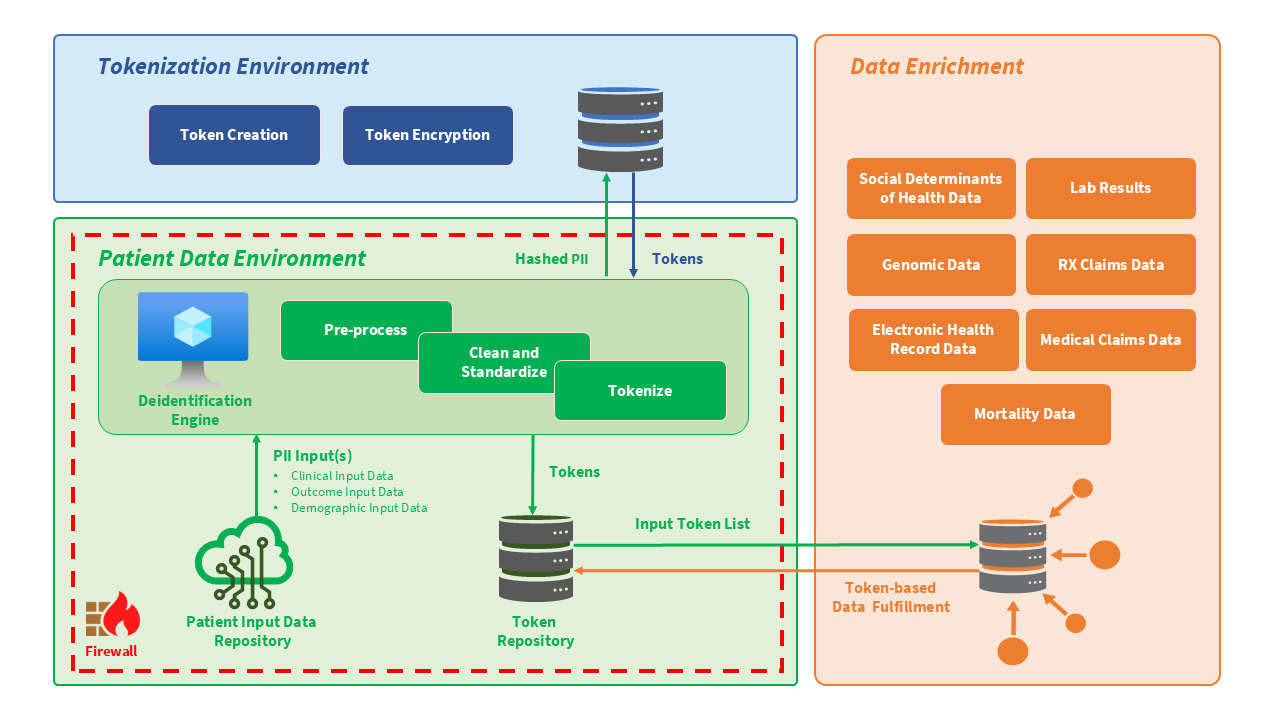

Supplement: Supplementary file 1 [file Image1.png]
